# The Impact of Chair–Team Sociodemographic Dissimilarity on the Relation Between Chair Power and Entrepreneurial Ventures’ R&D Intensity: Evidence From China

**DOI:** 10.3389/fpsyg.2020.603540

**Published:** 2021-02-09

**Authors:** Yaoyi Zheng, Shufen Dai, Yueting Li, Yi Su

**Affiliations:** ^1^School of Economics and Management, University of Science and Technology Beijing, Beijing, China; ^2^School of Economics and Management, Harbin Engineering University, Harbin, China

**Keywords:** board chair, power, R&D intensity, chair–team sociodemographic dissimilarity, entrepreneurship

## Abstract

Contemplating the actual leaders of entrepreneurial firms and socio demographic dissimilarity between leaders and their teams, this study adopts panel data on the entrepreneurial firms of the China’s Growth Enterprise Market and empirically examines the influence of chair power on research and development (R&D) intensity of entrepreneurial firms from the perspective of social identity. The results indicate that chair power positively affects entrepreneurial firms’ R&D intensity. The chair–team sociodemographic dissimilarity moderates the relationship in such a way that chair power is negatively related to entrepreneurial firms’ R&D intensity only when chair–team sociodemographic dissimilarity is high. The execution of robustness checks authenticates the veracity of the empirical results.

## Introduction

Leaders, as key decision-makers of enterprises, influence enterprise innovation, such as research and development (R&D) intensity, through using their power (Garms and Engelen, 2019; Ke and Li, 2020). To drive their business growth by exerting the innovative spirit of leaders to a greater extent, more and more entrepreneurial ventures follow some high-tech companies’ “power centralization model” to endow their leaders with many important positions, such as chairs, chief executive officers (CEOs), and chief technical officers (CTOs). However, the power model also has some risks. Leaders need to deeply understand whether the relationship between power intensity and entrepreneurial firms’ innovation is stable. Meanwhile, they also need to understand the potential boundary conditions under which power intensity affects entrepreneurial firms’ innovation.

Many studies have examined the impact of leader’s power on organizational decision-making and performance (e.g., Li and Jones, 2019; Vandekerkhof et al., 2019; Altunbaş et al., 2020; Tang et al., 2020). However, neither the corporate governance nor the organizational innovation literatures specially outline whether and how leader’s power influences enterprise innovation (Garms and Engelen, 2019; Ke and Li, 2020). Few relevant studies have focused on two aspects. On the one hand, how does the leader’s power directly affect firm innovation (e.g., Sariol and Abebe, 2017; Sheikh, 2018; Garms and Engelen, 2019; Ke and Li, 2020). This view points out that powerful leaders will influence strategic decision-making because of their preferences and then have impacts on enterprises innovation. On the other hand, what is the moderating mechanism of leader’s power on enterprise innovation? This view holds that the internal and external factors of the organization will affect the relationship between the leader’s power and the enterprise innovation. Existing research finds that the source of the leader (Sariol and Abebe, 2017), market competition (Sheikh, 2018), and legal environment (Ke and Li, 2020) are the important factors that affect the relationship between the leader’s power and enterprise innovation.

Combining with existing studies, there are still the following deficiencies. First, previous studies focus on the CEO/CTO power in large established corporations. These studies pay less attention to the entrepreneurial firms and their leaders, the chairs. For Chinese entrepreneurial firms, the legal representative are the chairs, who are the actual leaders and the most important decision-makers (Yang and Wang, 2014; Jiang et al., 2020). Compared with the large established corporations, entrepreneurial firms have low-resource endowment. And powerful chairs tend to have more far-reaching influence on entrepreneurial firms’ innovativeness by integrating the internal and external resources (Jiang et al., 2020). Therefore, the role of chair’s power in innovation activities of Chinese entrepreneurial firms cannot be ignored. Second, extant studies mainly analyze the economic consequences of leaders’ power according to agency theory and stewardship theory. These studies present the two opposite viewpoints—that is, powerful leaders play “self-interested role” and “housekeeper role.” The behavioral agency model, which combines the prospect theory and classic agency theory, emphasizes that the risk preference of decision-makers depends on different forms of monitoring. On the one hand, this model adds contextualized risk preferences to the classic agency model. On the other hand, it discusses the contextual factors that impact the risk decision-making behavior of the agents. In this regard, it not only makes up the deficiency of classic agency model, but also expands the research of prospect theory. Since its inception, the behavioral agency model has been widely used to explain the relationship between executive risk preferences and organizational outcomes (e.g., Martin et al., 2015; Poletti-Hughes and Briano-Turrent, 2019; Benischke et al., 2020). But this model used into the innovation research is still lacking. As the actual leaders of Chinese entrepreneurial enterprises, the chairs’ risk preferences under the specific monitoring context can be transformed into legitimate innovation activities such as increasing or decreasing investments in R&D, through the execution of power. Therefore, it is necessary to explore the influence of chairs’ power on the innovativeness of Chinese entrepreneurial enterprises by adopting the behavioral agency model.

Moreover, the examination of leader power on innovation activities of entrepreneurial firms would be incomplete without assessing the cross-level interaction between leaders and their teams. Previous studies suggest that the leaders’ personal characteristics in large established corporations play a significant role in affecting the relationship between leaders’ power and enterprise innovation (e.g., Pan et al., 2017; Sariol and Abebe, 2017). Nevertheless, strategic decisions of entrepreneurial firms are usually the result of the joint action of leaders and their teams (Ensley et al., 2002; Yang and Wang, 2014). Compared with the large established corporations, the external environment of entrepreneurial firms is more dynamic. The teams in entrepreneurial firms are required for undertaking extensive roles and complex tasks. A cross-level interaction between chairs and their teams in Chinese entrepreneurial firms will become particularly critical (Yang and Wang, 2014). Chair–team sociodemographic dissimilarity reflects that team members differ from the individual leader in age, gender, race, and other detectable demographic characteristics (Tsui et al., 1992; Georgakakis et al., 2017). This leads to differences in behavioral cognition, such as values and preferences between leaders and their teams, thus generating the diversified vertical interactions between leaders and their teams. This cross-level interaction between leaders and their teams caused by the differences in demographic characteristics will have an important impact on the decision-making behavior (Hambrick and Mason, 1984; Yang and Wang, 2014; Georgakakis et al., 2017). And it is particularly prominent in Chinese entrepreneurial firms whose decision-making core are the chairs and their teams, i.e., vice chairs, general managers, vice general managers, vice presidents, and chief financial officers. Therefore, it deserves further exploring whether the cross-level interaction caused by the social category dissimilarity between chairs and their teams affects the chairs’ risk preferences under the specific monitoring context and then impacts the innovation activities of entrepreneurial firms.

Against this backdrop, this study constructs the theoretical framework of chair power, chair–team sociodemographic dissimilarity, and R&D intensity of entrepreneurial firms and analyzes the interaction mechanism among them. It contributes to the ongoing scholarly on leader power and firm innovation in a few distinct ways. First, this study probes the relationship between chair power and R&D intensity of entrepreneurial firms. Extant studies focus on the CEO/CTO power and organizational innovation in large established corporations (e.g., Pan et al., 2017; Sariol and Abebe, 2017; Sheikh, 2018; Garms and Engelen, 2019; Ke and Li, 2020). Considering the sample characteristics of this study and the context of Chinese culture, this study empirically investigates the impact of chair power on the R&D intensity of entrepreneurial firms according to the behavioral agency model. It expands the perspective of leader power research based on enterprise innovation. It also provides empirical evidence for the integration of behavior perspective and innovation research. Second, the moderating effect of chair–team sociodemographic dissimilarity is investigated. Existing research highlights the role of leaders’ personal characteristics on the leaders’ power–innovation relationship in large established corporations (e.g., Sariol and Abebe, 2017; Pan et al., 2017). Relatively little is known on the important role of leaders and their teams’ functions in shaping the R&D investment strategies of entrepreneurial firms. This study explores the influence of cross-level interaction caused by chair–team sociodemographic dissimilarity on the relationship between chair power and R&D intensity of entrepreneurial firms. It provides new insights for upper echelons researchers.

In what follows, section “Theoretical Background and Hypotheses” explains the theoretical background and assumptions. Section “Materials and Methods” describes the research methodology and design, including sample selection, data sources, variable measurement indicators, and data analysis. Section “Results” reports the empirical results. Section “Discussion and Conclusion” presents a discussion of the research findings and their implications and outlines avenues for future research.

## Theoretical Background and Hypotheses

### Theoretical Background

This study mainly builds on the theories of behavioral agency model, social hierarchy theory, and social identity theory. The behavioral agency model combines prospect theory and classic agency theory, first proposed by Wiseman and Gomez-Mejia (1998). This model suggests that decision-makers are loss-averse rather than risk-averse. And the degree of risk preferences of decision-makers depends on the specific monitoring context they face. It reveals that problem-framing affects decision-makers’ risk perception and ultimately decides their risky choice. When the problem is framed as “positive,” the risk burden of decision-makers is increased. Decision-makers will reduce risk-taking in order to avoid the loss of their wealth. When a problem is framed as “negative,” decision-makers often take more aggressive risk behaviors to minimize the loss of their wealth. Behavioral agency model has been extensively used to explain decision-makers’ risk preferences and associated organizational performance since it is proposed (e.g., Martin et al., 2015; Poletti-Hughes and Briano-Turrent, 2019; Benischke et al., 2020). But this model used into the innovation research is still lacking. R&D investment has a high-risk nature. Decision-makers decide whether to invest resources in R&D by assessing the risks of innovation activities and considering the responsibilities they should be undertaking. For entrepreneurial firms, powerful chairs play a pivotal role in organizational resource allocation. Moreover, innovative activity is particularly important for enhancing their employment safety and maintaining their social status as powerful actors in the organizations (Jiang et al., 2020). According to this model, this study attempts to probe the influence of chair power on the R&D intensity of entrepreneurial firms.

To better explain the functional realization mechanism of decision-makers’ innovative strategic choice and the influence of the cognitive interaction between leaders and their teams, this study further introduces the social hierarchy theory and social identity theory. On the one hand, social hierarchy theory emphasizes that the ranking of individual members in social dimensions is the essential features of social hierarchy (Magee and Galinsky, 2008). Chinese enterprises are organized hierarchically (Shah et al., 2019b; Sarfraz et al., 2020c). The presence of the power distance and collectivist orientation in China keeps team members from violating the social hierarchy (Shao et al., 2013; Yang and Wang, 2014), which is beneficial for powerful chair in the high hierarchical positioning to improve the effective functioning of teams. And this is helpful for powerful chair to promote the formulation and implementation of innovative strategic decisions due to his/her multifaceted influences (Yang and Wang, 2014; Jiang et al., 2020). On the other hand, in the diversity literatures, team diversity is divided into two types, the social category diversity focusing on detectable attributes, such as age, gender, and ethnicity (Tsui et al., 1992), and the informational diversity focusing on less-visible and task-related attributes, such as educational specialization and functional background (Bantel and Jackson, 1989; Dayan et al., 2017). Social identity theory points out that dissimilarity in visible characteristics among team members can destroy team function. The reason is that individuals often categorize others based on observable demographic characteristics. And individuals will show the strong sense of identity with their “similar” members and exclude “different” members (Tsui et al., 2002). This creates a sense of estrangement and distance among team members, which eventually leads to the formation of “insiders” and “outsiders” within the team (Messick and Mackie, 1989). This state of interpersonal relationship will further affect the way they deal with information and knowledge from various parties. Therefore, this study focuses on detectable social category dissimilarity and further analyzes the moderating effect of chair–team social category dissimilarity on the relationship between chairs’ power and R&D intensity of entrepreneurial firms in combination with the above theories.

### Chair Power and Entrepreneurial Firms’ R&D Intensity

Finkelstein (1992) argues that the power intensity reflects individual’s ability to achieve his/her own will. And the leader power includes structural, ownership, expert, and prestige power according to the various sources of executive power. The studies based on large and mature enterprises have shown that leader’s power has a direct impact on enterprise innovation by transforming his/her own willing into legitimate innovation strategies (e.g., Sheikh, 2018; Garms and Engelen, 2019; Jiang et al., 2020). And for many entrepreneurial firms whose development is driven by innovation, the strategic decision-making is particularly critical. The leaders of entrepreneurial firms exercise power to consolidate their dual roles as the core of the team and the core of organizational decision-making. They participate in and execute the key strategies of entrepreneurial firms, including the R&D intensity.

R&D intensity usually refers to the degree an enterprise creates new products and knowledge by increasing R&D investment (Choi and Williams, 2014). And it also reflects the investment and implementation degree of innovation strategies. The investment period of enterprise innovation is long and unpredictable. And it is usually accompanied with uncertainty of future earnings (Paik and Woo, 2017). Accordingly, leaders are less likely to pursue the R&D path in order to protect their existing wealth. However, leaders of entrepreneurial firms are more adventurous relative to the general managers (Su et al., 2019). In this regard, the enhancement of power intensity is beneficial to the chairs of entrepreneurial firms to convert their preferences into legalized organizational behavior. In addition, according to the behavior agent model, whether the resources are allocated to R&D depends on the problem-framing of decision-makers. And the final decision is guided by their risk perception under the specific monitoring context they face. This study contends that powerful chairs of entrepreneurial firms tend to pursue enterprise’s R&D investment. First, powerful leaders who are committed to consolidating their role as the executors of shaping the organizations’ direction are more likely to view R&D investment as “loss aversion” strategic decisions. Although R&D investment carries a high level of uncertainly in their payoffs, the formulation and implementation of these strategic decisions provide powerful leaders with the greatest opportunity to enhance their employment security and social status. Second, R&D investment can be perceived as a beneficial source of long-term competitiveness. It can promote the development of entrepreneurial ventures toward large and highly diversified enterprises. In this way, leaders’ authority can be consolidated, and stakeholders’ support can be continuously obtained. Third, powerful leaders are more optimistic about risks and pay more attention to the potential returns of risks (Altunbaş et al., 2020; Zhao et al., 2020). This may contribute to their stronger tendency to invest in R&D, thus effectively enhancing the R&D intensity of enterprises.

Powerful chairs can promote the formulation and implementation of innovation strategies with their own influence. First, a social hierarchy governs the ranking of individual members in social dimensions (Magee and Galinsky, 2008; Sarfraz et al., 2020c). In this regard, the hierarchical positions of individual members within organizations indicate their social status (Shah et al., 2019b; Sarfraz et al., 2020c). Chinese enterprises are organized hierarchically (Shah et al., 2019a,b). And there is a distinct hierarchy between chairs and their team members in entrepreneurial firms. The high power distance typical of Chinese culture makes powerful chair in the apex of the team effectively ameliorate intrateam conflicts and power struggles. It helps the chair to standardize the decision-making process of the team and expedite intragroup cooperation and coordination (Smith et al., 2006; Halevy et al., 2011). These interactive processes have the critical driving effect on creative–innovative decision-making (Dayan et al., 2017). It helps the teams to develop R&D strategies, ultimately transforming into R&D investment of enterprises. Second, powerful chairs usually play multiple roles and have more internal and external influence (Yang and Wang, 2014; Jiang et al., 2020). This helps to strengthen their power in the decision-making of R&D strategy. In addition, according to the resource-based view, resources play a key role in the development of innovation activities. Powerful leaders who can better integrate the internal and external resources play powerful roles in enterprise innovation activities (Park and Tzabbar, 2016; Ke and Li, 2020). This helps promote the innovative use of resources (Sheikh, 2018), which is particularly important for entrepreneurial firms with low-resources endowment to implement the innovation-driven development model. As such, the following hypothesis is presented:

***Hypothesis 1****: Chair power is positively related to the R*&*D intensity of entrepreneurial firm in China.*

### Moderating Effect of Chair–Team Sociodemographic Dissimilarity

The chair–team sociodemographic dissimilarity reflects that each member of the team is different from the chair in age, gender, and other detectable attributes. According to the social identity theory, individuals will scrutinize and evaluate themselves by comparing observable demographic characteristics with other individuals. When individuals find that they have similar characteristics with other individuals, differences between “in-group” and “out-group” will be formed, leading to in-group favoritism and out-group hostility (Messick and Mackie, 1989; Shin et al., 2012). The cross-level sociodemographic dissimilarity between chairs and their teams due to social categorization will affect the interaction between chairs and their teams. It will also destroy the construction of good psychological state and interpersonal relationship between leaders and their teams and impede the process of innovative strategic decision-making (Li T. et al., 2020). The similarity-attraction paradigm can also explain the negative effect of the chair–team sociodemographic dissimilarity from another perspective. According to this perspective, similarity of detectable demographic characteristics among individuals can form a strong sense of attraction, promoting communication between individuals. Dissimilarity of detectable demographic characteristics among individuals will reduce this attraction, leading to less interaction between individuals (Georgakakis et al., 2017). Following this view, one might expect that the chair–team sociodemographic dissimilarity will stimulate the formation of self-categorization, which in turn affects the construction of the relationship between leaders and their teams (Liden et al., 1996).

The destruction of social process between leaders and their teams will further affect the development of task process. The cross-level sociodemographic dissimilarity of chairs reflects the diversity among members. Thus, it may provide different knowledge for R&D strategic decisions. Past research suggests that the informational diversity would be beneficial because of the wider range of task-relevant resources brought by dissimilar participants, whereas social category diversity would be detrimental to the effective functioning of teams due to the formation of “insiders” and “outsiders” within the teams (e.g., Bantel and Jackson, 1989; Shin et al., 2012; Dayan et al., 2017). Consequently, we argue that the chair–team sociodemographic dissimilarity would destroy the orderly progress of social process such as good interpersonal communication between leaders and their teams. Meanwhile, the destruction of this good social process between leaders and their teams will further affect the development of task process such as the teams’ cross-level processing of information from various parties. Specifically, the leader–team sociodemographic dissimilarity damages the psychological identify between the leader and other team members, thus debilitating information exchange and knowledge integration (Yoshida et al., 2014; Georgakakis et al., 2017). On the one hand, the leader–team sociodemographic dissimilarity is likely to keep team members from identifying with the leader and thus from establishing intrateam identification (Yoshida et al., 2014). The suppression of leader–team identification often disrupts cross-subgroup integration and fails to unlock the knowledge potential of team diversity (Hoever et al., 2012). On the other hand, externally visible demographic dissimilarities between the leader and the rest of team reduce the ability of the former to act as a bridge builder for facilitating informational exchange (Georgakakis et al., 2017). The ability is important for a leader, whose role is to promote effective elaboration using team’s task-relevant information (Mitchell et al., 2015) and use it to shape creative and innovative decision-making to positively influence the R&D intensity of entrepreneurial firm.

Unlike the production and business behavior, R&D investment is a high-risk activity that requires comprehensive and high-quality strategic formulation in the early stage. Because of the market position and development stage of entrepreneurial enterprises, the innovation elements and knowledge acquired by entrepreneurial enterprises are limited. The leaders and their teams are the strategic decision-making body of entrepreneurial firms. And when facing the complex R&D investment decision-making, the cross-level interaction and identification between the leaders and their teams are more needed (Yang and Wang, 2014). In this way, information exchange and sharing can be better promoted, heterogeneous resources of team knowledge can be fully exploited, and more comprehensive intellectual support can be provided for R&D investment decisions. Although powerful chairs can promote innovative strategic decision-making, this study contends that the formation of cross-level sociodemographic dissimilarity will reduce the interaction effects between leaders and their teams through two approaches: social process and task process. It damages the quality of innovative strategic decision-making, which in turn impairs the chairs’ cognitive ability on enterprise innovation. It is the cognition that enables the leader to execute innovative strategies that pave the way toward investment in R&D (Sarfraz et al., 2020a). Consequently, we argue that the risk-taking of the chairs would be increased in these cases. According to the behavior agent model, to preserve their wealth and enhance their social status, powerful chairs are more likely to utilize the internal and external influence to attenuate the R&D intensity of entrepreneurial firms. The above arguments lead to the following hypothesis:

***Hypothesis 2:*** *The chair–team sociodemographic dissimilarity moderates the positive effect of chair power on R&D intensity of entrepreneurial firm such that the positive effect is lessened when chair–team sociodemographic dissimilarity is higher.*

## Materials and Methods

### Sample and Data Construction

Given that China’s Growth Enterprises Market (CGEM) was officially opened on October 23, 2009, the firms listed before December 31, 2011, in CGEM are selected. And the listed firms that existed before 2003 are removed. Taking 2012–2016 as the research period, a total of 80 firms and 320 firm–year combinations were obtained. Those firms whose board chairs are changed and their annual reports without R&D expenditure data are further excluded. This leads to the selection of an eligible sample of 75 firms and 300 firm–year combinations. We set 2016 as the latest year for the sample enterprises. On the one hand, this allows meeting the definition of entrepreneurial ventures (Paik and Woo, 2017). On the other hand, this allows meeting the research needs by obtaining the detailed information for multiple years. To avoid single-year sampling bias (Amason et al., 2006) and examine the dynamic effects of chair power on entrepreneurial firms’ R&D intensity, a longitudinal dataset has been developed. Specifically, a 1 year lag period (year *t* to *t* + 1) is used. This study focuses on public organizations. First, CGEM has low threshold and convenient financing, which is favored by many innovative entrepreneurial firms (Su et al., 2019). The phenomenon of chair power concentration in these firms is prominent, which are appropriate for the research purposes. Second, they provide an efficient source of detailed information for multiple years (Amason et al., 2006). Third, top managers and power hierarchies are well-articulated in public organizations; the fit between chair power and innovativeness can be assumed to be better.

Much of the data are taken from the China Stock Market and Accounting Research (CSMAR) database. The missing values from the CSMAR database are sought using the WIND database and annual reports. The consistency of multisource data is checked. And the annual reports shall prevail in case of discrepancy. The data for demographic characteristics and power structure of chairs and their teams are hand-collected from multiple sources, including the CSMAR database, annual reports, and corporate websites.

### Measures

#### R&D Intensity

R&D intensity is measured as the ratio of R&D expenditures to total assets. Entrepreneurial firms often have very low and unstable sales, and whose total assets are stable relative to established firms (Paik and Woo, 2017). Their inclusion may lead to biased R&D intensity normalized by sales, as some companies may have high R&D intensity because of their limited sales rather than because of their extensive R&D investment. Thus, we standardize R&D investment by total assets rather than normalize R&D expenditures by firm sales to measure the R&D intensity of entrepreneurial firms. The measurement has strong reproducibility and stability relative to the subjective measurement. Hence, it has been applied by many strategic management researchers (e.g., Paik and Woo, 2017; Garms and Engelen, 2019).

#### Chair Power

Existing studies usually focus on the CEO/CTO in organizations. However, for Chinese listed company, the legal representative is the board chair, who is the actual leader and the most important decision-maker (Yang and Wang, 2014; Jiang et al., 2020). Thus, choosing the chair as the “leader” to perform the function of strategic formulation is more in line with China’s reality and the sample characteristics of this study.

Power in top managers as proposed by Finkelstein (1992) includes structural, ownership, expert, and prestige power. This approach is usually adopted by extant research. Nevertheless, when the specific measures are taken, prestige power is usually not to be considered (e.g., Sariol and Abebe, 2017; Sheikh, 2018). Unlike the studies in the Western context, leaders with prestige power are usually more influential in society, and more accessible to social capital in the Chinese context. And this is particularly important for those entrepreneurial firms that are trying to obtain scarce resources for R&D activities. Therefore, considering the sample characteristics of this study and the actual situation of China, prestige power is included in the measurement of chair power.

In this study, chair power is operationalized as the composite measure including structural power (chair duality), ownership power (chair shareholding, chair founder status), expert power (chair title), and prestige power (chair political connections). Chair duality is operationalized as a binary variable taking the value of 1 if the chair also serves as a CEO and 0 otherwise. Chair shareholding is also operationalized as a binary variable taking the value of 1 if the chair holds shares of the firm and 0 otherwise. Chair founder status is operationalized as a binary variable with a value of 1 if the chair is the founder or cofounder in team members and 0 otherwise. Chair title is operationalized as a binary variable with a value of 1 if the chair has high-level technical title in the industry and 0 otherwise. Chair political connections as a binary variable taking the value of 1 if the chair has political connections with the governments and 0 otherwise. Similar to the study of Sheikh (2018), chair power is constructed by summing each dimension to come up with the final variable. Higher scores indicate greater power.

#### Chair–Team Sociodemographic Dissimilarity

This variable is a composite of a chair’s dissimilarity to the rest of the team in terms of two externally observable sociodemographic attributes: age and gender. The sociodemographic attributes include age, gender, and ethnicity (Tsui et al., 1992; Georgakakis et al., 2017). Considering that ethnic characteristics in studies based on Chinese background are not as significant as those in countries such as the United States, this study focuses on gender and age. As gender is a categorical variable, gender dissimilarity between the chair and the team (vice chairs, general managers, vice general managers, vice presidents, and chief financial officers) is calculated using a modified version of Blau’s (1977) formula expressed as (1*- P*_*ikt*_)^2^, where *P*_*ikt*_ is the proportion of the chair *i* for *k* organization in *t* year share the same gender category with team members. Chair–team age dissimilarity is measured using the distance formula: ${\left[\sum_{j=1}^{n-1}{\left(x_{i\,k\,t}-x_{j\,k\,t}\right)}^{2}\,/\,n-1\right]}^{1/2}$ (Westphal and Zajac, 1995), where *x*_*ikt*_ is the age of chair *i* for *k* organization in *t* year, *x*_*jkt*_ is the age of executive *j* (excluding the chair) for *t* year in *k* company, and n is the number of team members. Following Georgakakis et al. (2017), we rescale the age dissimilarity to take values between 0 and 1 and then aggregated the two components in a composite variable. This variable is averaged to create an indicator of chair–team sociodemographic dissimilarity.

#### Control Variables

Similar to the prior studies (Yang and Wang, 2014; Dayan et al., 2017; Sheikh, 2018; Ke and Li, 2020; Su and Li, 2020), several variables that might influence R&D intensity are controlled. The variable on individuals is chair’s gender, coded as 1 for a woman and 0 for a man. These variables on teams are team size, measured as the number of top managers; chair–team educational specialty dissimilarity, measured using the formula: (1*- P*_*ikt*_)^2^, where *P*_*ikt*_ is the proportion of the chair *i* for *k* organization in *t* year share the same educational specialty category with team members, and chair–team functional background dissimilarity, measured using the formula: (1*- P*_*ikt*_)^2^, where *P*_*ikt*_ is the proportion of the chair *i* for *k* organization in *t* year, share the same functional background category with team members. Educational specialty is categorized as science, engineering, economics, management, literature or art, law, or others. Functional background is categorized as manufacturing, R&D, finance or accounting, public relations, legal, literature or art, management, or others. At the organizational level, the variables are firm size, measured as the natural logarithm of total assets; asset–liability ratio, measured by the ratio of total liabilities to total assets; percentage of stock, measured by shareholding ratio of top 10 shareholders; ROE, measured as the ratio of net profits to net assets; cash ratio, measured as the proportion of corporate monetary funds and marketable securities to current liabilities; and turnover, measured as the natural logarithm of organizational turnover. Industry and year dummies are included to eliminate the potential impact of unobservable time and industry heterogeneities (Paik and Woo, 2017).

### Analytical Approach

Given that the R&D intensity variable whose value ranges from 0.001 to 0.235 is a censored variable with a zero lower bound, tobit regression rather than zero-inflated regression is used to test the study’s predictions (Paik and Woo, 2017; Protogerou et al., 2017; Jeong and Shin, 2019). Moreover, as the study’s independent variable, chair power is composed of dimensions such as the following: chair founder status is time invariant, and a fixed-effects approach is inappropriate for handling this data. And the null hypotheses of the Hausman test are accepted suggesting that fixed-effects estimation is not appropriate for the model. Accordingly, a random-effects tobit regression is used in the analysis.

## Results

### Main Results

Table 1 exhibits descriptive statistics and pairwise correlations for the study variables. Although the variance inflation factors (VIFs) are not reported separately, this study computes them to identify any multicollinearity concerns in regression analyses. All the VIF values are less than 3.5, indicating that there are no indications of multicollinearity in our study due to the small VIF values.

**TABLE 1:** Descriptive statistics and correlations.

													
**Variables**	**1**	**2**	**3**	**4**	**5**	**6**	**7**	**8**	**9**	**10**	**11**	**12**	**13**
1. Chair’s gender	1.000												
2. Team size	–0.002	1.000											
3. Dissimilarity of chair–team education	−0.156***	−0.316***	1.000										
4. Dissimilarity of chair–team function	–0.013	−0.259***	0.904***	1.000									
5. Firm size^a^	0.033	0.236***	−0.114**	–0.043	1.000								
6. Asset–liability ratio	–0.072	−0.110*	0.120**	0.192***	0.123**	1.000							
7. Percentage of stock	−0.287***	−0.123**	0.084	0.027	0.129**	0.091	1.000						
8. ROE	–0.024	–0.026	0.017	0.009	0.041	0.133**	0.198***	1.000					
9. Cash ratio	–0.001	–0.016	–0.074	−0.101*	0.036	–0.091	0.090	−0.109*	1.000				
10. Turnover^a^	–0.083	0.216***	0.052	0.078	0.072	0.048	–0.047	0.298***	−0.350***	1.000			
11. Sociodemographic dissimilarity	−0.356***	0.164***	–0.027	–0.029	0.094	0.026	0.046	0.035	−0.127**	0.220***	1.000		
12. Chair power	−0.161***	–0.017	0.221***	0.194***	0.014	0.040	0.216***	0.078	0.064	0.042	–0.076	1.000	
13. R&D intensity	–0.082	0.109*	–0.081	−0.122**	0.128**	0.015	0.047	0.150***	0.007	–0.021	–0.002	0.066*	1.000
Mean	0.053	7.207	0.350	0.360	18.466	0.509	0.613	0.060	0.040	10.908	0.546	3.283	0.032
*SD*	0.225	2.152	0.259	0.250	5.231	0.988	0.113	0.067	0.107	0.839	0.132	1.155	0.034
Min	0.000	3.000	0.000	0.020	7.395	0.015	0.212	–0.114	0.001	8.600	0.044	0.000	0.001
Max	1.000	15.000	1.000	1.000	25.725	6.781	0.817	0.282	1.293	12.882	0.799	5.000	0.235

n = 300. ^*a*^Log-transformed. ***P < 0.01, **P < 0.05, *P < 0.1.

Table 2 shows the panel random-effects tobit regression analysis results. To observe incremental changes in variance explained across different stages of the analysis, the control variables were first entered, and then the independent variable, moderator variables, and interaction effects were added in subsequent models. By contrast with ordinary least square (OLS) panel regression, random-effects tobit regression does not report within *R*^2^ or adjusted *R*^2^, whereas goodness-of-fit of the models can be observed by logarithmic likelihood function. The value of the log likelihood in Table 2, from Model 1 to Model 4, is increased. Thus, the variables added in different stages can effectively improve the overall explanatory strength of the model.

**TABLE 2:** Panel random-effects tobit regression analysis results of entrepreneurial firms’ R&D intensity.

**Variables**	**Baseline regressions**		**Effect of chair–team social dissimilarity**		**Supplementary analysis**	
	**Model 1**	**Model 2**	**Model 3**	**Model 4**	**Model 5**	**Model 6**
Chair’s gender	−0.0241*	−0.0213	−0.0250*	−0.0244*	−0.0200	−0.0120
	(−1.79)	(−1.58)	(−1.73)	(−1.69)	(−1.45)	(−0.85)
Team size	−0.0009	−0.0011	−0.0010	−0.0010	−0.0010	−0.0010
	(−0.87)	(−1.02)	(−0.95)	(−0.92)	(−0.96)	(−0.99)
Dissimilarity of chair–team education	0.0187	0.0174	0.0146	0.0150	0.0191	0.0211
	(0.84)	(0.79)	(0.65)	(0.67)	(0.86)	(0.96)
Dissimilarity of chair–team function	−0.0379*	−0.0390*	−0.0370*	−0.0372*	−0.0414*	−0.0401*
	(−1.70)	(−1.75)	(−1.66)	(−1.66)	(−1.82)	(−1.78)
Firm size	0.0004	0.0002	0.0003	0.0003	0.0002	0.0003
	(0.57)	(0.37)	(0.44)	(0.39)	(0.35)	(0.47)
Asset–liability ratio	−0.0012	−0.0013	−0.0014	−0.0015	−0.0013	−0.0014
	(−0.52)	(−0.58)	(−0.61)	(−0.65)	(−0.60)	(−0.63)
Percentage of stock	−0.0059	−0.0102	−0.0117	−0.0114	−0.0097	−0.0068
	(−0.27)	(−0.47)	(−0.53)	(−0.52)	(−0.44)	(−0.31)
ROE	0.0911***	0.0893***	0.0885***	0.0882***	0.0887***	0.0841***
	(3.40)	(3.37)	(3.34)	(3.33)	(3.35)	(3.19)
Cash ratio	−0.0070	−0.0093	−0.0106	−0.0111	−0.0102	−0.0115
	(−0.40)	(−0.53)	(−0.60)	(−0.63)	(−0.58)	(−0.66)
Turnover	−0.0033	−0.0033	−0.0032	−0.0030	−0.0033	−0.0039
	(−1.16)	(−1.20)	(−1.12)	(−1.05)	(−1.19)	(−1.42)
Industry (year) dummies	Yes	Yes	Yes	Yes	Yes	Yes
Chair power		0.0051**	0.0051**	0.0052**	0.0049**	0.0055**
		(2.37)	(2.34)	(2.41)	(2.24)	(2.53)
Chair–team social dissimilarity			−0.0144	−0.0227	−0.0003	−0.0005
			(−0.72)	(−1.00)	(−0.51)	(−0.91)
Chair power × chair–team social dissimilarity				−0.0104		−0.0010**
				(−0.76)		(−2.37)
Log likelihood	671.561	674.397	674.657	674.947	674.529	677.300
Wald *χ*^2^	58.71***	64.25***	64.92***	65.80***	64.34***	71.53***

n = 300; z-value in parentheses. ***P < 0.01, **P < 0.05, *P < 0.1.

All the hypotheses were tested. As shown in Table 2 (Model 2), chair power is positively related to R&D intensity of entrepreneurial firms (*r* = 0.0051, *P* < 0.05). Hypothesis 1 is thereby confirmed. Model 4 reveals that chair–team socio demographic dissimilarity has an insignificant moderating effect on the relationship between chair power and R&D intensity of entrepreneurial firm (*r = -*0.0104, *P >* 0.1). Hypothesis 2 is thereby not confirmed.

### Supplementary Analysis

The above results indicate that chair–team sociodemographic dissimilarity insignificantly moderates the relationship between chair power and R&D intensity of entrepreneurial firm. The possible reason is that the leader in entrepreneurial firm exhibits more risk-seeking behaviors relative to the general managers (Su et al., 2019), thus weakening the impact of gender difference between the chair and the team. This further reduces the impact of chair–team sociodemographic dissimilarity. To verify this hypothesis, we remove gender characteristics and construct chair–team sociodemographic dissimilarity by using age characteristics. The results are shown in Table 2. Model 6 of Table 2 reveals that chair–team sociodemographic dissimilarity has a significant moderating effect on the relationship between chair power and R&D intensity of entrepreneurial firm (*r* = −0.0010, *P* < 0.05), thus supporting Hypothesis 2. In addition, other results remain qualitatively similarly.

### Robustness Checks

Several robustness checks were conducted to further verify the findings. First, corporate ownership is one of the important differences among Chinese companies (Yang and Wang, 2014; Li H. et al., 2020; Sarfraz et al., 2020b). Considering the influence of corporate ownership, we excluded the 5 years data corresponding to a state-owned enterprise in the sample and conducted regression for non-state-owned enterprises. Model 7 in Table 3 shows that chair power significantly impacts R&D intensity (*r* = 0.0061, *P* < 0.01). Chair–team sociodemographic dissimilarity negatively moderates the relationship between chair power and R&D intensity (*r* = −0.0012, *P* < 0.01). And both coefficient value and significance level are improved. This indicates that the results are more prominent in non-state-owned enterprises.

**TABLE 3:** Robustness checks.

**Variables**	**Considering the corporate ownership**	**Using alternative definition of chair power**	**Chair-team sociodemographic dissimilarity replaced with team-level diversity**	**The curvilinear effect of chair–team social dissimilarity**
	**Model 7**	**Model 8**	**Model 9**	**Model 10**
Chair’s gender	−0.0105	−0.0249*	−0.0195	−0.0160
	(−0.75)	(−1.73)	(−1.44)	(−1.05)
Team size	−0.0006	−0.0006	−0.0011	−0.0010
	(−0.60)	(−0.54)	(−1.08)	(−0.91)
Dissimilarity of chair–team education	0.0227	0.0140	0.0167	0.0218
	(1.06)	(0.62)	(0.76)	(0.98)
Dissimilarity of chair–team function	−0.0379*	−0.0340	−0.0372*	−0.0418*
	(−1.73)	(−1.47)	(−1.67)	(−1.85)
Firm size	0.0003	−0.0000	0.0003	0.0003
	(0.47)	(−0.03)	(0.41)	(0.42)
Asset–liability ratio	−0.0011	−0.0008	−0.0012	−0.0014
	(−0.52)	(−0.35)	(−0.55)	(−0.61)
Percentage of stock	−0.0088	−0.0081	−0.0071	−0.0075
	(−0.42)	(−0.37)	(−0.32)	(−0.34)
ROE	0.0780***	0.0871***	0.0819***	0.0863***
	(3.10)	(3.30)	(3.04)	(3.26)
Cash ratio	−0.0095	−0.0130	−0.0108	−0.0108
	(−0.57)	(−0.74)	(−0.61)	(−0.61)
Turnover	−0.0046*	−0.0045	−0.0036	−0.0038
	(−1.73)	(−1.61)	(−1.30)	(−1.36)
Industry (year) dummies	Yes	Yes	Yes	Yes
Chair power	0.0061***	0.0044*	0.0053**	0.0051**
	(2.85)	(1.76)	(2.49)	(2.34)
Chair−−team social dissimilarity	−0.0006	−0.0010	−0.0013	−0.0011
	(−1.07)	(−1.41)	(−0.04)	(−0.65)
Chair power × chair–team social dissimilarity	−0.0012***	−0.0010**	−0.0396	
	(−2.88)	(−2.20)	(−1.44)	
Chair–team social dissimilarity^2^				0.0000
				(0.39)
Chair power × chair–team social dissimilarity^2^				−0.0001
				(−1.63)
Log likelihood	681.444	675.736	675.436	675.955
Wald *χ*^2^	70.77***	66.24***	67.06***	68.00***
Observations	296	300	300	300

z-value in parentheses. ***P < 0.01, **P < 0.05, *P < 0.1.

To further investigate the impact of chair power on R&D intensity of entrepreneurial enterprises, chair power was measured from the cross-level perspective. Chair duality is operationalized as a binary variable taking the value of 1 if the chair also serves as a CEO and 0 otherwise. Chair shareholding is also operationalized as a binary variable taking the value of 1 if the chair holds shares of the firm at a higher rate than other members and 0 otherwise. Chair founder status is operationalized as a binary variable with a value of 1 if the chair is the sole founder relative to other members and 0 otherwise. Chair title is operationalized as a binary variable with a value of 1 if the chair has high-level technical title in the industry while other members do not and 0 otherwise. Chair political connections as a binary variable taking the value of 1 if the chair has political connections with the governments while other members do not and 0 otherwise. Similar to the study of Sheikh (2018), chair power is constructed by summing each dimension to come up with the final variable. Higher scores indicate greater power. Model 8 in Table 3 shows that chair power significantly impacts R&D intensity (*r* = 0.0044, *P* < 0.1). Chair–team sociodemographic dissimilarity negatively moderates the relationship between chair power and R&D intensity (*r* = −0.0010, *P* < 0.05).

One could speculate that team-level compositional diversity may influence the functioning of chair power in the way as chair–team dissimilarity. To assess this, the regressions were re-evaluated with chair–team sociodemographic dissimilarity replaced with team-level diversity. Model 9 of Table 3 shows that team-level social dissimilarity shows a non- significant moderating effect (*r* = −0.0396, *P* > 0.1).

To address the possibility that too much sociodemographic dissimilarity may has positive effects through generating social norms (Tsui et al., 2002). We assessed the curvilinear effect of chair–team sociodemographic dissimilarity by creating an interaction term involving chair power and the square of chair–team sociodemographic dissimilarity indicator. Model 10 of Table 3 shows that this term is not significant (*r* = −0.0001, *P* > 0.1).

A fifth test shows that the association between chair power and R&D intensity of entrepreneurial firm is actually stronger in technology-intensive industries than that in non-tech-intensive industries (capital-intensive and labor-intensive industries) (*r* = 0.0067 vs. *r* = 0.0040). Considering that tech-intensive industries requires high R&D intensity, this finding further supports the core argument that empowering more directed at the chairs becomes particularly effective.

Finally, reverse causality may be a concern (e.g., Paik and Woo, 2017; Sarfraz et al., 2020a); that is, chair power of entrepreneurial firm may be affected by the firm’s greater R&D intensity rather than vice versa. The power index is composed of five different sources of power. Some of these sources such as chair stock ownership, chair titles, and political connections evolve over a period of time and are related to firm innovation. Chairs that invest more in R&D usually have higher stock ownership, higher technical titles, and more political connections. Thus, chairs commit greater resource to R&D and may be more powerful. If we do not consider these reverse causalities, then our chair power effect may be systematically biased.

This problem was addressed by two ways. On the one hand, following Fisman and Svensson (2007), Sheikh (2018), and Ke and Li (2020), yearly industry region (province) average power index is used as the instrumental variable of chair power. The Hausman specification test and Durbin–Wu–Hausman test are, respectively, used to test the endogeneity of chair power. The results of Hausman test show that the statistic *χ*^2^(1) is 1.85, and the *P*-value is 0.174. It indicates that there is no endogenous explanatory variable. Durbin–Wu–Hausman test results show that statistic *χ*^2^(1) is 2.09, and the *P*-value is 0.147. The result also indicates that chair power is not an endogenous explanatory variable. Moreover, GMM C statistic *χ*^2^(1) is 1.79, and the *P*-value is 0.180. Thus, the above test results show that there is no obvious endogeneity problem.

On the other hand, drawing on the method of Belderbos et al. (2014), the possible reverse causality between chair power and R&D intensity is tested. Chair power in period *t* + 1 is taken as the dependent variable, and the R&D intensity of entrepreneurial enterprise in period *t* is taken as the independent variable. The regression results show that the coefficient of R&D intensity is not significant (*r* = 0.5802, *P* > 0.1). Similarly, chair power in period *t* is taken as the dependent variable, and the R&D intensity of entrepreneurial enterprise in period *t* is taken as the independent variable. The results remain unchanged (*r* = −0.0076, *P* > 0.1), which further helps to exclude the possibility of reverse causality.

## Discussion and Conclusion

Unlike large established corporations, does chair power promote the R&D intensity of entrepreneurial firms? How does chair–team sociodemographic dissimilarity in entrepreneurial firms affect the above relationship? The discussion of these issues is conducive to extend the current understanding of the impact leaders’ power has on the enterprises innovation. By adopting panel data on the entrepreneurial firms of China’s Growth Enterprise Market, the results reveal a significant positive relationship between chair power and entrepreneurial firms’ R&D intensity. Furthermore, chair–team sociodemographic dissimilarity attenuates the positive effect of chair power on R&D intensity of entrepreneurial firms. By changing the measurement method of key variables, testing the influence of non-linear effects, and controlling other factors, the veracity of the above findings is authenticated.

The conclusion of this study provides a systematic theoretical logic to explain how chairs’ power affects R&D intensity of entrepreneurial firms under the context of China. The theoretical contributions of this study are twofold. First, prior studies investigate the impact of CEO/CTO power on the innovativeness of large established corporations (e.g., Sariol and Abebe, 2017; Sheikh, 2018; Garms and Engelen, 2019; Ke and Li, 2020). By considering the governance structure of Chinese entrepreneurial firms and the context of Chinese culture, the current study probes the influence of chair power on the R&D intensity of entrepreneurial firms. This expands the perspective of the research on the leader’s power in the direction of enterprise innovation. Further, the study explains the innovation-driven effect of chair power based on behavioral agency model, which provides empirical evidence for the integration of behavioral perspective and innovation research.

Second, extant studies focus on the moderating effect of leaders’ personal characteristics on the relationship between leaders’ power and innovation in large established corporations (e.g., Pan et al., 2017; Sariol and Abebe, 2017). This study analyzes the role of chair–team sociodemographic dissimilarity on the chairs’ power–R&D intensity relationship by considering the decision-making body of Chinese entrepreneurial firms. The finding confirms the importance of the cross-level social category dissimilarity of chair when the relationship between chair power and entrepreneurial firm’s R&D intensity is investigated. Accordingly, this study expands the contextual effect of the behavioral agency model. It also provides new insights for upper echelons researchers. The current study focuses specifically on the dissimilarity between chairs and their teams and its moderating role in Chinese entrepreneurial firms. The results, both for the effects of chair–team dissimilarity and the *post hoc* null findings regarding the effects of team-level compositional diversity, highlight that, at least in certain situations (e.g., entrepreneurial firms led by powerful chairs), the moderating effect of team composition on chair influence may derive mostly from the relative differences between the chair and her/his team. Thus, distinguishing chairs from their teams may prove fruitful in advancing our overall understanding of the joint influence of the chairs and their teams.

This study has a number of implications for research. First, by investigating the relationship between chairs’ power and innovation activity of Chinese entrepreneurial firms, this study extends the current understanding of power that chairs have on the organizations they lead. As such, the findings of this study improve scholars’ understanding by providing empirical evidence on the level of risky R&D investment of Chinese entrepreneurial firms that powerful chairs are more likely to pursue. Second, this research also illustrates the link between powerful chairs and their cross-level sociodemographic dissimilarity that influences R&D intensity of Chinese entrepreneurial firms. These findings also hold practical implications that include monitoring of executives appointment and cross-level management of teams in entrepreneurial firms. The positive relationship between chairs’ power and R&D intensity suggests that entrepreneurial firms that seek investment in R&D should be open to the idea of endowing their chairs with greater power to foster their innovative spirit. Specifically, the appointment of the chair in entrepreneurial firm should follow the principle that the chair has political connections with the governments, belongs to the founder, and has high-level technical title in the industry. In addition, the chair should hold shares and serve as a CEO and other important positions in entrepreneurial firms. Further, the results of robustness checks also provide insights into the importance of corporate ownership and industry categories. Specifically, these findings suggest that the innovation-driven effects of chairs’ power are more prominent in non-state-owned entrepreneurial firms. And empowering more directed at the chairs in entrepreneurial firms of tech-intensive industries becomes particularly effective. Furthermore, the findings of this study also provide insights into the importance of chair–team social category dissimilarity. Specifically, when making appointment decisions such as external introduction, internal promotion, and intergenerational inheritance, we should pay attention to the composition of observable demographic characteristics of leaders and their teams in addition to considering the person-post matching. By reducing the chair–team sociodemographic dissimilarity, especially the age differences between chairs and their teams in Chinese entrepreneurial firms, it can exert the innovative spirit of powerful leaders to a greater extent.

As with any research, this study has some limitations. First, power is regarded as a comprehensive and unified whole, ignoring the heterogeneity in different power dimensions. For example, managers’ power can be divided into power depth and power breadth according to the multiple power sources (Garms and Engelen, 2019). The functional mechanism of these two kinds of power may be different. Future research can be continued from the perspective of power heterogeneity. Second, this study uses demographic indicators as proxies for behavioral cognition. Although this approach is supported by theories and lots of empirical evidence, it still has defects (Yang and Wang, 2014). Methods such as qualitative comparative analysis can be used in future studies to capture the vertical interaction process between leaders and their members. Finally, because of data limitations, we perform the analysis on the entrepreneurial firms of the China’s Growth Enterprise Market. Future studies should examine the innovation-driven effect of chair power using a much larger sample (e.g., adding non-publicly entrepreneurial firms) to enhance the external validity of the findings.

## Data Availability Statement

The raw data supporting the conclusions of this article will be made available by the authors, without undue reservation.

## Ethics Statement

The studies involving human participants were reviewed and approved by School of Economics and Management at University of Science and Technology Beijing. The patients/participants provided their written informed consent to participate in this study.

## Author Contributions

All authors contributed to the article and approved it for publication.

## Conflict of Interest

The authors declare that the research was conducted in the absence of any commercial or financial relationships that could be construed as a potential conflict of interest.
